# The Potato Nucleotide-binding Leucine-rich Repeat (NLR) Immune Receptor Rx1 Is a Pathogen-dependent DNA-deforming Protein*

**DOI:** 10.1074/jbc.M115.672121

**Published:** 2015-08-25

**Authors:** Stepan Fenyk, Philip D. Townsend, Christopher H. Dixon, Gerhard B. Spies, Alba de San Eustaquio Campillo, Erik J. Slootweg, Lotte B. Westerhof, Fleur K. K. Gawehns, Marc R. Knight, Gary J. Sharples, Aska Goverse, Lars-Olof Pålsson, Frank L. W. Takken, Martin J. Cann

**Affiliations:** From the ‡ School of Biological and Biomedical Sciences,; § Biophysical Sciences Institute,; ** Department of Chemistry, Durham University, South Road, Durham DH1 3LE, United Kingdom,; the ¶ Laboratory of Nematology, Department of Plant Sciences, Wageningen University, 6708 PB, Wageningen, The Netherlands, and; ‖ Molecular Plant Pathology, Swammerdam Institute for Life Sciences, University of Amsterdam, Science Park 904, 1098 XH, Amsterdam, The Netherlands

**Keywords:** cellular immune response, DNA-binding protein, host-pathogen interaction, Nod-like receptor (NLR), plant biochemistry, plant defense, plant virus

## Abstract

Plant nucleotide-binding leucine-rich repeat (NLR) proteins enable cells to respond to pathogen attack. Several NLRs act in the nucleus; however, conserved nuclear targets that support their role in immunity are unknown. Previously, we noted a structural homology between the nucleotide-binding domain of NLRs and DNA replication origin-binding Cdc6/Orc1 proteins. Here we show that the NB-ARC (nucleotide-binding, Apaf-1, R-proteins, and CED-4) domain of the Rx1 NLR of potato binds nucleic acids. Rx1 induces ATP-dependent bending and melting of DNA *in vitro*, dependent upon a functional P-loop. *In situ* full-length Rx1 binds nuclear DNA following activation by its cognate pathogen-derived effector protein, the coat protein of potato virus X. In line with its obligatory nucleocytoplasmic distribution, DNA binding was only observed when Rx1 was allowed to freely translocate between both compartments *and* was activated in the cytoplasm. Immune activation induced by an unrelated NLR-effector pair did not trigger an Rx1-DNA interaction. DNA binding is therefore not merely a consequence of immune activation. These data establish a role for DNA distortion in Rx1 immune signaling and define DNA as a molecular target of an activated NLR.

## Teaser

**Background:** Direct targets for plant NLR proteins in immune signaling are largely unknown.

**Results:** The Rx1 NLR protein of potato binds and distorts DNA following pathogen perception, resulting in immune activation.

**Conclusion:** DNA is a direct signaling target for a plant NLR immune receptor.

**Significance:** Plant NLR receptors might regulate immune transcriptional responses by directly interacting with plant chromatin.

## Introduction

Plants and animals possess innate immune systems enabling individual cells to mount a defense response upon pathogen perception (1–4). The NLR^3^ family immune receptors perceive non-self and modified self molecules inside host cells and mediate immune responses to invading microorganisms. Plant NLRs typically detect strain-specific pathogen effectors, whereas the animal NLRs commonly recognize microbe- or damage-associated molecular patterns (3, 5, 6). The NLR families in both kingdoms belong to the STAND P-loop ATPases of the AAA+ superfamily, whose multidomain structure allows them to function simultaneously as sensor, switch, and response factor (7, 8).

Plant NLRs are named after their central NB and C-terminal LRR domains. The N terminus is highly divergent; in plants, this region typically encompasses CC or TIR domains (3). The NB domain of plant NLRs is commonly referred to as the NB-ARC domain and has been proposed to function as a molecular switch (8–10). The LRR confers pathogen recognition specificity and maintains the NLR protein in a signaling-competent yet autoinhibited state. Biochemical analysis of tomato I-2 and Mi-1, flax M and L6, and barley MLA27 revealed that the NB-ARC domain is ADP-bound in the autoinhibited state (11–13). LRR-mediated pathogen recognition is proposed to permit the exchange of ADP for ATP, allowing the NB-ARC domain to adopt an activated or “on” state. ATP hydrolysis to ADP enables the “off” state to be re-established. Support for this model comes from studies where I-2 mutants defective in ATP hydrolysis *in vitro* are autoactivated *in vivo* and from an autoactive flax M mutant that preferentially co-purifies with ATP (12, 13). Recently, the NB subdomain of rice Os2g_25900 and NB-ARC domains of maize pollen-signaling protein (PSiP) and *Arabidopsis* Rpm1 were demonstrated to possess a nucleotide phosphatase activity compatible with the switch model (14).

Activation of animal NLRs typically triggers NB domain-mediated self-association, resulting in the formation of a cytoplasmic signaling scaffold on which partners are activated due to their induced proximity (15). For plant NLRs, such partners have not been identified, and a pivotal yet unanswered question concerns the nature of the downstream signaling component(s) and how these are activated by NLR proteins in their “on” state. The identity of the specific NLR subdomain that transduces a signal to such a downstream component is also unresolved. Whereas for Rx1, the NB subdomain of Rx1 induces cell death, the N-terminal TIR domains of L6 and RPS4 or the coiled-coil domain of MLA10 suffices to trigger cell death, suggesting that the signaling domain might vary for different NLRs or that they act as heterodimers (11, 16–18). The location of the NLR signaling event is also the subject of increased scrutiny. Several NLR proteins, including N, Mla10, and Rx1 have a dynamic nuclear-cytoplasmic distribution, whereas RRS1-R is restricted to the nucleus, dependent upon the presence of the PopP2 immune elicitor (19–23). Genetic screens for compromised NLR-mediated resistance identified genes encoding components of the nuclear pore complex (24), indicating involvement of nuclear transport in immune signaling. More direct proof for nuclear activity is the observed nuclear localization for barley MLA1 and MLA10, *Arabidopsis* RPS4 and SNC1, and the tobacco N protein (22, 25–27). Redirection of nucleus resident MLA10, N, RPS4, and SNC1 to the cytoplasm compromises their ability to activate immune signaling, suggesting a nuclear signaling target (19, 22, 26, 28). The potato Rx1 protein, which confers PVX resistance, localizes to both cytoplasm and nucleus (23). The Rx1 N terminus interacts with a member of the RanGAP2 family that controls nuclear-cytoplasmic trafficking through the nuclear pore (29). Together, these studies indicate that nuclear-cytoplasmic trafficking and compartmentalization are essential for NLR protein function and suggest distinct activities in different cellular compartments. Recent studies on *Arabidopsis* RPS4 and barley Mla10 (25, 30) have shown that induction of cell death is associated with cytoplasmic localization, whereas nuclear localization of RPS4 is associated with local resistance responses. The presence of a WRKY DNA-binding domain in RRS1-R (21) and the association of Mla10 with both Myb and WRKY transcription factors (31) have led to the hypothesis that plant NLRs regulate transcription in the immune response (32). This notion is further supported by interactions between an SPL transcription factor and the tobacco N NLR protein, the interaction between the SNC1 NLR protein of *Arabidopsis* and the TPR1 transcriptional co-repressor, and the presence of BED DNA-binding domains in many plant NLRs (27, 33–35).

Based on these observations, signaling from plant NLRs can be viewed from two perspectives that are not necessarily mutually exclusive. In the first perspective, activated NLRs may act as platforms from which signaling proteins promoting immune responses are permitted to function. Alternatively, NLRs may themselves have an additional biochemical activity, independent of their ATPase activity, required for direct activation of plant immunity. In support of the latter model, we here demonstrate that the Rx1 NLR protein of potato is able to bind DNA *in vitro* and *in situ* and that its *in vitro* activity consists of bending and melting DNA. We further demonstrate that the interaction of Rx1 with DNA as observed *in situ* only occurs after its genuine activation by the coat protein of PVX virus.

## Experimental Procedures

### 

#### 

##### Structural Modeling

Protein fold searches using the Phyre^2^ protein homology/analogy recognition engine version 2.0 (36) were undertaken using amino acids 143–488 of Rx1, using both normal and intensive modeling modes. Similar structural homology was also detected using the SAM-T08, Hidden Markov Model-based protein structure prediction server (37). All superpositions were performed using the SSM algorithm in Coot (38). Models of Rx1 based on Cdc6/Orc1 (PDB accession number 2V1U) were made using Chainsaw within the CCP4 package (39), and sequence alignments were generated by the Phyre^2^ server. Side chain packing and energy minimization was performed using GalaxyRefine (40). Figures were generated using the PyMOL molecular graphics system (41).

##### Plasmids

A PCR product spanning residues 1–489 of Rx1 (GenBank^TM^ accession number AJ011801.1) was cloned into the NcoI and BamHI sites of pET32c (pET32c-Rx1(1–489)) and fitted with a hexahistidine tag for affinity purification of recombinant protein. The oligonucleotides used to construct pET32c-Rx1(1–489) were 5′-GCC CCA TGG CTT ATG CTG CTG TTA C-3′ (sense) and 5′-GGC GGA TCC TTA TGC ACA TGA ATT TTG ATC ACT C-3′ (antisense). Mutant constructs were generated by site-directed mutagenesis. A PCR product corresponding to amino acids 177–339 of PSiP (Gene ID 542027) was cloned into the XhoI and NcoI sites of pRSET-B (pRSET-PSiP(177–339)) and fitted with a hexahistidine tag for affinity purification of recombinant protein. The primers used to construct pRSET-PSiP(177–339) were 5′-GGC CTC GAG AAA GGC TGT GGG TGG CCT TG-3′ (sense) and 5′-GGC CCA TGG TCA CTT GAT TGC ACA ATA ATG CCC A-3′ (antisense). A PCR product corresponding to amino acids 1–126 of histone H2B of *Arabidopsis thaliana* (locus AT3G09480) was subcloned into the Gateway entry vector pDONR207 via BP recombinant reaction and transferred via LR reaction into the plant binary vector pK7WGF2 (pK7WGF2-H2B) to fuse the open reading frame (ORF) to an N-terminal green fluorescent protein (GFP) ORF. The primers used to construct pK7WGF2-H2B were 5′-GGG GAC AAG TTT GTA CAA AAA AGC AGG CTA CAA CAA TGG CCATG GCA CCG AAG GCA GAG-3′ (sense) and 5′-GGG GAC CAC TTT GTA CAA GAA AGC TGG GTC AGA ACT GGT GAA TTT GGT G-3′ (antisense). pBIN-35S-based plasmids corresponding to NBARC-GFP, CC-NBARC-GFP, NBARC-LRR-GFP, GFP-LRR, CC-GFP, Rx1-GFP, GFP-NLS-Rx1, GFP-NES-Rx1, CP105, and CP106 are as described (23). Mutant constructs were generated by site-directed mutagenesis. Pto and AvrPto were expressed using a construct that contains 35S promoter-driven *Pto* and *avrPto* as described (42). For the construction of Rx1-4Strep, a double STREPII tag (43) (-asWSHPQFEKggWSHPQFEKts-) was created by annealing the oligonucleotidesm-Str1 (5′-GGC CGC TAG CTG GAG TCA CCC TCA GTT CGA GAA GGG TGG ATG GTC ACA TCC ACA ATT TGA AAA GAC TAG TTA AT-3′) and m-Str2 (5′-CTA GAT TAA CTA GTC TTT TCA AAT TGT GGA TGT GAC CAT CCA CCC TTC TCG AAC TGA GGG TGA CTC CAG CTA GC-3′) and ligating the annealed oligonucleotides between the NotI and XbaI of pRAP 35S:YFP-myc (23), replacing the sequence encoding the Myc tag. From the resulting pRAP::YFP-STR2, a 4-fold STREPII tag was generated by fusing the AscI-SpeI 35S::YFP-STR2 with the NheI-PacI STR2-Tnos segment in pRAP digested with AscI-PacI. In the resulting pRAP::YFP-STR4 vector, GFP was replaced by Rx1 cDNA using the NcoI and NotI sites as described for pRAP:Rx-GFP (23). The expression cassette was excised using the AscI and PacI restriction sites and introduced into the expression vector pHYG (44). The expression vector pHYG-Rx1-4Strep was transformed to *Agrobacterium tumefaciens* strain MOG101 for plant expression.

##### Protein Expression and Purification

Protein corresponding to the NB-ARC domain of PSiP (amino acids 178–505; PSiP-NBARC) was generated as described previously (14).

A 10-ml culture of pET32c-Rx1(1–489) (Rx1(1–489) wild type and mutant proteins) in *Escherichia coli* C41(DE3) was grown overnight in Luria broth supplemented with 100 μg ml^−1^ ampicillin at 37 °C. This culture was diluted into 1 liter of Luria broth supplemented with 100 μg ml^−1^ ampicillin and grown at 37 °C to *A*_600 nm_ = 0.7. The growth temperature was reduced to 22 °C, and growth continued to *A*_600 nm_ = 1.0. Protein production was induced at 22 °C for 16 h with 100 μm isopropyl-β-d-thiogalactoside. Pelleted cells were washed with 50 mm Tris-HCl, pH 8.5, 1 mm EDTA, and the pellet was resuspended in twice its volume of 50 mm Tris-HCl, pH 7.5, 100 mm NaCl, 5 mm EDTA. Cells were centrifuged (2700 × *g*, 30 min, 10 °C), and the pellet was resuspended in twice its volume of 50 mm Tris-HCl, pH 7.5, 100 mm NaCl, 5 mm EDTA, 1% (v/v) Triton X-100. Cells were lysed by sonication (150 s) and centrifuged (75,500 × *g*, 60 min, 10 °C), and inclusion bodies were washed twice in 5 ml of 50 mm Tris-HCl, pH 8.0, 100 mm NaCl, 5 mm EDTA, 1% (v/v) Triton X-100. The final pellet was resuspended in 2 ml of 50 mm Tris-HCl, pH 9.0, 100 mm NaCl, 1 mm EDTA, 1 mm dithiothreitol, 8 m urea). Material was incubated at 50 °C for 20 min prior to centrifugation (20,000 × *g*, 30 min, 15 °C) and the pellet was discarded. The supernatant was dialyzed into 50 mm Tris-HCl, pH 8.5, 100 mm NaCl, 7 m urea and incubated with Ni^2+^-nitrilotriacetic acid resin (Qiagen) for 60 min at 4 °C. Resin was washed with 10 bed volumes of buffer A (50 mm Tris-HCl, pH 7.5, 400 mm NaCl, 20 mm MgCl_2_, 0.25 mm imidazole, 7 m urea), 10 bed volumes of buffer B (buffer A with 1.5 m NaCl, 20 mm imidazole), and 20 bed volumes of buffer C (buffer B with 10 mm NaCl). Protein was eluted with 5 bed volumes of elution buffer (buffer C with 200 mm imidazole). Protein was resuspended at 1 mg ml^−1^ in 50 mm Tris-HCl, pH 8.5, 9.6 mm NaCl, 0.4 mm KCl, 2 mm MgCl_2_, 2 mm CaCl_2_, 0.5 m arginine, 0.4 m sucrose, 0.75 m guanidine HCl, 1 mm glutathione, 0.1 mm reduced glutathione and incubated at 4 °C for 1 h. Refolded protein was dialyzed into 20 mm Tris-HCl, pH 7.5, 50 mm NaCl, 2 mm MgCl_2_, loaded onto a monoQ anion exchange column (GE Healthcare), and eluted using a 50 mm to 1 m NaCl gradient. Peak fractions containing Rx1(1–489) protein were concentrated; dialyzed into 20 mm Tris-HCl, pH 7.5, 150 mm NaCl, 2 mm MgCl_2_; and loaded onto a Superdex 200 gel filtration column, and peak fractions were eluted in the same buffer.

pRSET-PSiP(177–339) (PSiP-NB) was expressed in *E. coli* BL21(DE3) *cya*::*kan* at 22 °C for 16 h with 100 μm isopropyl-β-d-thiogalactoside. Pelleted cells were washed with 50 mm Tris-HCl, pH 7.5, 1 mm EDTA resuspended in 50 mm Tris-HCl, pH 7.5, 250 mm NaCl, 100 μm EDTA and lysed by sonication (150 s), and the supernatant was incubated with Ni^2+^-nitrilotriacetic acid resin for 60 min at 4 °C. Resin was washed with 10 bed volumes of buffer D (50 mm Tris-HCl, pH 7.5, 400 mm NaCl, 5 mm imidazole, 100 μm EDTA), buffer E (buffer D with 1.5 m NaCl and 20 mm imidazole), and buffer F (buffer E with 40 mm NaCl). Protein was eluted with 5 bed volumes of buffer F containing 200 mm imidazole. PSiP-NB was subsequently purified by anion exchange chromatography as described previously (14). Orc1-1 and Orc1-3 of *Sulfolobus solfataricus* were expressed and purified as described previously (45).

For Rx1-4Strep, *A. tumefaciens* strain MOG101 was transformed with construct pHYG-Rx1-4Strep and grown to *A*_600 nm_ of 1.0 in 20 g liter^−1^ sucrose, 5 g liter^−1^ Murashige and Skoog basal salt mixture, 1.95 g liter^−1^ MES, pH 5.6, 200 μm acetosyringone. The two youngest fully expanded leaves of 5–6-week-old *Nicotiana benthamiana* plants were infiltrated completely. Infiltration was performed by injecting the *Agrobacterium* suspension into a *N. benthamiana* leaf at the abaxial side using a 1-ml syringe. Leaf material was harvested after 48 h and ground in liquid nitrogen with a mortar and pestle. Ground material was resuspended 1:10 (w/v) in 10% (v/v) glycerol, 50 mm Tris-HCl, pH 8.5, 150 mm NaCl, 2 mm MgCl_2_, 0.1% (v/v) Tween 20, 5 mm DTT, 0.02 g ml^−1^ polyvinylpolypyrrolidone, 0.2 mg ml^−1^ Pefabloc SC protease inhibitor (Roche Applied Science). Cell debris and polyvinylpolypyrrolidone were removed by centrifugation (20,000 × *g*, 20 min, 4 °C). The extract was passed through a Sephadex G25 column, and the flow-through was supplemented with 5 mm DTT, 0.2 mg ml^−1^ Pefabloc SC protease inhibitor, and 20 μg ml^−1^ avidin. The extract was incubated with Streptactin superflow resin (IBA) at 4 °C overnight. Resin was washed with 10 bed volumes wash buffer (10% (v/v) glycerol, 50 mm Tris-HCl, pH 8.5, 150 mm NaCl, 2 mm MgCl_2_, 0.1% (v/v) Tween 20, 5 mm DTT). Protein was eluted with 2 bed volumes of wash buffer supplemented with 15 mm desthiobiotin. Purified protein was dialyzed into 20 mm Tris-HCl, pH 7.5, 150 mm NaCl, 2 mm MgCl_2_ before use.

##### Circular Dichroism

80 μm protein was dialyzed into double-distilled H_2_O at 4 °C. The baseline CD spectra of blank sample (double-distilled H_2_O) and 1.7 μm protein were measured using a J-810 spectropolarimeter (Jasco) at 180–300 nm (20 nm min^−1^). The averaged data of replicate blank spectra were subtracted from the protein spectra, and the data were normalized to zero at 250 nm. The corrected CD spectra from 190–240 nm were analyzed using CDPro (46). The protein database generating the lowest root mean square deviation was used as the best approximation for secondary structure content.

##### Electrophoretic Mobility Shift Assays

The oligonucleotides used for quantitative EMSA are derived from a series of oligonucleotides that enables a comparison of relative DNA binding affinity to varying DNA topologies independent of DNA sequence (47). The oligonucleotides sequences were 5′-TGG GTC AAC GTG GGC AAA GAT GTC CTA GCA ATG TAA TCG TCT ATG ACG TT-3′ (SS1; DNA sense strand), 5′-AAC GTC ATA GAC GAT TAC ATT GCT AGG ACA TCT TTG CCC ACG TTG ACC CA-3′ (SS2; DNA antisense strand), and 5′-UGG GUC AAC GUG GGC AAA GAU GUC CUA GCA AUG UAA UCG UCU AUG ACG UU-3′ (RNA sense strand) (47). Oligonucleotides were end-labeled with 10 μCi of [γ-^32^P]ATP using T4 polynucleotide kinase, and unincorporated nucleotides were removed using Micro Bio-Spin columns (Bio-Rad). Protein and 0.15 nm nucleic acids (oligonucleotide 1 ssDNA, annealed oligonucleotide 1 and oligonucleotide 2 dsDNA, and ssRNA) were incubated in 20 mm Tris-HCl, pH 8.0, 60 mm NaCl (unless otherwise stated), 2 mm EDTA, 1 mm DTT, 10% (v/v) glycerol, 0.1 mg/ml BSA for 20 min on ice. Quantitative EMSAs were separated on a native 7% (w/v) polyacrylamide gel. Experiments to assess the role of nucleotides in DNA binding used binding reactions and gels supplemented with 10 mm ZnCl_2_ and nucleotide. Polyacrylamide gels were dried and analyzed by autoradiography. EMSAs using unlabeled virion DNA were separated using 0.8% (w/v) Tris acetate-EDTA-agarose gels and stained with ethidium bromide. All reported values for *K_d_* represent apparent *K_d_* due to the potential for dissociation of protein-DNA complexes during electrophoresis. Curves were fitted by non-linear regression in GraphPad Prism version 6.0.

##### Construction of DNA Structures

DNA substrates corresponding to double-stranded branched structures (F12-ds/ds), branch structures with two single-stranded arms (F12-ss/ss), and branch structures with one double-stranded and one single-stranded arm (F12-ds/ss) were made by annealing synthetic oligonucleotides from a series that enables the comparison of relative DNA binding affinity to varying DNA topologies independent of DNA sequence (47). Oligonucleotide sequences were 5′-GAC GCT GCC GAA TTC TGG CTT GCT AGG ACA TCT TTG CCC ACG TTG ACC C-3′ (SS3), 5′-GCC AGA ATT CGG CAG CGT C-3′ (LAG), and 5′-AAC GTC ATA GAC GAT TAC A-3′ (LEAD). SS3 was end-labeled with 10 μCi of [γ-^32^P]ATP using T4 polynucleotide kinase, and unincorporated nucleotides were removed using Micro Bio-Spin columns (Bio-Rad). SS3, SS1, LAG, and LEAD were annealed to make F12-ds/ds. SS3, SS1, and LEAD were annealed tomake F12-ds/ss. SS3 and SS1 were annealed to make F12-ss/ss. SS3 and the corresponding antisense oligonucleotide (5′-GGG TCA ACG TGG GCA AAG ATG TCC TAG CAA GCC AGA ATT CGG CAG CGT C-3′) were annealed to make a linear dsDNA control (dsF12) and SS3 used as linear ssDNA control (ssF12). Annealing synthetic oligonucleotides with a defined sequence mismatch made DNA substrates corresponding to linear DNA containing bubbles of defined length. Oligonucleotide sequences were 5′-TTT GGT CTA ACT TTA CCG CTA CTA AAT GCC GCG GAT TGG TTT CGC TGA ATC AGG TTA TTA-3′ (P1), 5′-TAA TAA CCT GAT TCA GCG AAA CCA ATC CGC GGC ATT TAG TAG CGG TAA AGT TAG ACC AAA-3′ (P2), 5′-TAA TAA CCT GAT TCA GCG AAC CAA TCG CAA CCA TTT AGT AGC GGT AAA GTT AGA CCA AA-3′ (P5), 5′-TAA TAA CCT GAT TCA GCG AAA CAT TGT AGG TAA GCT TAG TAG CGG TAA AGT TAG ACC AAA-3′ (P6), and 5′-TAA TAA CCT GAT TCA GCG AAT GAC CGA TAA CGT CCA CTT GAG CGG TAA AGT TAG ACC AAA-3′ (P7). P1 was end-labeled with 10 μCi of [γ-^32^P]ATP using T4 polynucleotide kinase, and unincorporated nucleotides were removed using Micro Bio-Spin columns. P1 and P2 were annealed to make linear dsDNA (dsP1). P1 and P5 were annealed to make linear dsDNA with a 5-nucleotide bubble (dsP1-5). P1 and P6 were annealed to make linear dsDNA with a 13-nucleotide bubble (dsP1-13). P1 and P7 were annealed to make linear dsDNA with a 20-nucleotide bubble (dsP1-20). P1 was used on its own as a ssDNA control (ssP1). All substrates were gel-purified on a native 10% (w/v) polyacrylamide gel. EMSA was performed as described above.

##### ATPase Assays

ATPase assays were typically performed at 37 °C for 30 min with 2.3 μm protein in 50 mm Bistris propane, pH 7.5, 10 mm MgCl_2_, and 5 μm ATP. Reactions were spiked with 0.5 μCi of 2,8-^3^H-labeled ATP for quantitation. Reactions were spotted onto a silica thin layer chromatography plate with 1 mm ADP to act as marker and carrier. The plates were developed in isobutyl alcohol/3-methyl-1-butanol/2-ethoxyethanol/ammonia/H_2_O (9:6:18:9:15). Spots were visualized at 256 nm and quantified using an AR-2000 TLC scanner.

##### Time-resolved FRET in Vitro

Synthetic oligonucleotides, unlabeled or end-labeled with fluorescein or tetramethylrhodamine, were purchased from Eurofins MWG. The oligonucleotides used were 5′-TGG GTC AAC GTG GGC AAA GA-3′ (sense strand) and 5′-TCT TTG CCC ACG TTG ACC CA-3′ (antisense strand). Strands were annealed by heating to 90 °C for 3 min in 10 mm Tris, pH 8.0, 1 mm EDTA before cooling to room temperature. Measurements used 1.5 μm protein with 50 nm DNA in the presence of 60 mm NaCl and were incubated for 10 min at room temperature before analysis. Time-resolved FRET was assessed using the time-correlated, single photon counting technique. The excitation source was a Picoquant pulsed diode laser LDH-P-C-485 (excitation wavelength 485 nm, 70-ps pulse full width at half-maximum at 20 MHz). Fluorescence was detected using an avalanche photodiode (Id Quantique 100-50) linked to a Becker and Hickl SPC 130 time-correlated, single photon counting module. An instrument response function of ∼200 ps was measured from Rayleigh scattered light. Fluorescence decays were collected for both donor- and donor-acceptor-labeled double-stranded DNA with or without protein using band pass filter detection of the donor emission and at magic angle polarization. Data were analyzed by the Grinvald-Steinberg method (48) to obtain the fluorescence lifetime for the donor and acceptor (τ_DA_)- and donor only (τ_D_)-labeled oligonucleotides. The data were fitted to a sum of exponentials using an iterative least squares reconvolution procedure with the optical/electrical excitation profile to produce a biexponential decay containing two lifetimes. This profile was obtained from a slide covered with silica LUDOX® particles, which provides an instant scatter of the excitation pulse. This data-fitting method provided more accuracy in the determination of shorter lifetimes than calculating a single average lifetime. Donor-acceptor distances (*R*) were calculated using the equation, *E* = *R*_0_^6^/(*R*_0_^6^ + *R*^6^), and a calculated Förster distance (*R*_0_) of 49.99 Å. The total length of the oligonucleotide with linkers and fluorescent dyes, at maximum extension, was calculated as 81.1 Å.

##### P_1_ Nuclease Sensitivity

Oligonucleotides for P_1_ nuclease sensitivity were 5′-CTC AAT ACA ATT GTC TCT GTG TAA ATT TCC TAC GTT TCA TCT GAA AAT CTA GCT ATT AGA GCT TGG TTT A-3′ (sense strand) and 5′-TAA ACC AAG CTC TAA TAG CTA GAT TTT CAG ATG AAA CGT AGG AAA TTT ACA CAG AGA CAA TTG TAT TGA G-3′ (antisense strand) and represent the C3/mORB dual site sequence at *oriC2* of *S. solfataricus* (49). The sense strand oligonucleotide was end-labeled with 10 μCi of [γ-^32^P]ATP as described above, and sense and antisense oligonucleotides were annealed as required. Reactions were performed in 20-μl volumes containing 20 mm Tris acetate, pH 7.5, 10 mm magnesium acetate, 100 mm NaCl, 0.15 nm oligonucleotide, and 1.5 μm protein. Protein was allowed to bind for 10 min at 37 °C. P_1_ nuclease was added to a final concentration of 0.01–0.1 units μl^−1^ and incubated for a further 20–60 min at 37 °C. Reactions were stopped with 5 μl of 100 mm Tris-HCl, pH 8.0, 2.5% (w/v) SDS, 100 mm EDTA, 10 units μl^−1^ proteinase K. 5 μl of loading buffer (97.5% (v/v) formamide, 10 mm EDTA, 0.3% (w/v), 0.3% bromphenol blue) was added, and reactions were electrophoresed on a 15% (w/v) polyacrylamide gel with 8 m urea. Polyacrylamide gels were dried and analyzed by autoradiography.

##### Time-resolved FRET in Situ

*A. tumefaciens* strain GV3101 (pMP90) was transformed with constructs pK7WGF2(GFP negative control), pK7WGF2-H2B (GFP-H2B positive control), pBIN35S-NBARC-GFP, pBIN35S-CC-NBARC-GFP, pBIN35S-NBARC-LRR-GFP, pBIN35S-GFP-LRR, pBIN35S-CC-GFP, pBIN35S-Rx1-GFP, pBIN35S-CP105, or pBIN35S-CP106 and grown to *A*_600 nm_ 0.8 in YEB medium supplemented with 20 μm acetosyringone and 10 mm MES, pH 5.6. Cells were washed three times in infiltration medium (10 mm MES, pH 5.6, 2% (w/v) sucrose, 20 μm acetosyringone) and infiltrated at *A*_600 nm_ 0.4 into 4–5-week-old *N. benthamiana* leaves. Leaves were harvested after 72 h and prior to any observed cell death in a compatible immune interaction, and the agroinfiltrated region was infiltrated with 10 μg/ml LDS 751 (Molecular Probes, Inc.). For experiments with CP105 and CP106, the elicitor-encoding *A. tumefaciens* culture was infiltrated into preinfiltrated sectors after 48 h (24 h before harvest). Leaves were fixed for 4 h at room temperature in 4% (w/v) paraformaldehyde in phosphate-buffered saline (PBS). Fixative was quenched for 30 min at room temperature in 125 mm glycine, and leaves were washed in PBS at 4 °C before mounting. A modified Zeiss Axiovert inverted epifluorescence microscope was used for time-resolved fluorescence microscopy. The overall excitation/detection of the fluorescence was performed using the time-correlated single photon counting technique. The excitation source was a Picoquant pulsed diode laser LDH-P-C-440 (excitation wavelength 440 nm, 70-ps pulse full width at half-maximum at 20 MHz). The objective lens (Zeiss ×100 oil immersion Ph3) focused the excitation light on the sample material. The emission was detected using suitable band pass/long pass filters for GFP and LDS 751 fluorescence, respectively. Fluorescence was detected with a photon counting module (Id Quantique 100-50) in a single photon counting mode. Data fitting was performed as for time-resolved FRET *in vitro*. The relative orientation of the GFP tag does not affect Rx1 function (23); nor does it affect the ability to observe energy transfer. All data are reported for the analysis of GFP lifetimes because LDS 751 emission is influenced by photobleaching and variability in concentration.

##### Statistical Analysis

*Error bars* represent the S.E. with the number of replicates as indicated in the figure legends. Statistical comparisons (*p* values) for data that pass a test for normality (D'Agostino and Pearson omnibus normality test and Shapiro-Wilk normality test) were obtained from one-way ANOVA with the indicated post hoc test. Statistical comparisons (*p* values) for data that do not pass a test for normality were obtained from a Kruskal-Wallis test with a post hoc multiple comparisons test. *p* values in statistical comparisons are indicated in the figures through *letters* and indicate compared data sets as described in the figure legends.

## Results

### 

#### 

##### Plant NLRs Are Structurally Related to Cdc6/Orc1 Family Proteins

**FIGURE 1.**
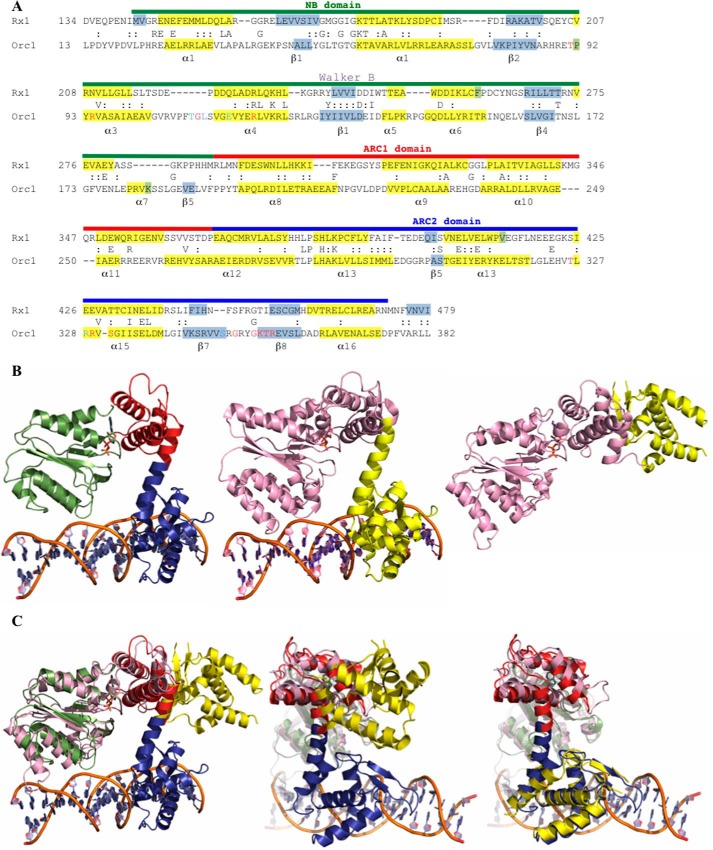
**Structural modeling of the Rx1 NB-ARC domains.** *A*, alignment of Rx1 (residues 134–479) with Orc1 of *A. pernix* (PDB code 2V1U; residues 13–382). *Numbers* denote amino acid residue position. Sequences are in standard single-letter amino acid code, and functionally related residues between the two proteins are indicated by a *colon*. The Rx1 domain structure is denoted by a *colored line above* the Rx1 sequence and corresponds to the NB (*green*), ARC1 (*red*), and ARC2 (*blue*) domains. Residues in *light blue* contact DNA bases in the Orc1-DNA structure, whereas those in *red* contact DNA bases and/or the DNA backbone (45, 49). Known (Orc1) and predicted (Rx1) secondary structures (α-helix (*yellow*) or β-sheet (*gray*)) are indicated. *B*, structural homology model for Rx1 based on the crystal structure of DNA-bound Cdc6/Orc1 from *A. pernix. Left*, structural homology model of the NB-ARC domain of Rx1 (amino acids 143–4780, with associated ADP (NB domain (*green*), ARC1 domain (*red*), or ARC2 (*blue*)). *Center*, crystal structure of *A. pernix* Cdc6/Orc1 in complex with DNA (PDB 2V1U) (*pink*, amino acids 13–279; *yellow*, amino acids 280–399). *Right*, crystal structure for Cdc6/Orc1 of *P. aerophilum* not bound to DNA (PDB code 1FNN) (*pink*, amino acids 1–278; *yellow*, amino acids 279–388). *C*, comparison of the PDB 2V1U-based Rx1 homology model with the crystal structure of Cdc6/Orc1 of *P. aerophilum* (PDB code 1FNN). *Left*, complete overlay of both structures. Note that only the NB (*green*) and ARC1 (*red*) superimposes and that the ARC2 domain (*blue*) of Rx1 is rotated compared with the C-terminal region of Cdc6/Orc1 of *P. aerophilum* (*yellow*). *Center*, overlay highlighting the C-terminal ARC1 (*red*) and ARC2 (*blue*) domains of Rx1. *Right*, superposition of the C-terminal domain of Cdc6/Orc1 of *P. aerophilum* onto the Rx1 model. Domain designations are as in *B*.

The *Rx1* gene, introgressed in potato from the wild species *Solanum andigena*, confers resistance to PVX upon recognition of its coat protein (50, 51). The Rx1 protein is a member of the CC-NB-LRR class of plant NLR proteins that consists of an N-terminal CC domain, a central NB-ARC domain, and a C-terminal LRR domain. The NB domain, containing a central β-sheet flanked by α-helices, is flanked by two ARC subdomains. ARC1 forms a four-helix bundle, and ARC2 adopts a winged helix fold characteristic of DNA-binding transcription factors (52). We hypothesized that an investigation of proteins structurally related to the Rx1 NB-ARC domain could provide insight into NLR biochemistry. Amino acids 143–488, encompassing the NB-ARC domain, were analyzed using the Phyre^2^ protein fold recognition engine and expected matches with the pro-apoptotic proteins CED-4 (PDB code 2A5Y) and Apaf-1 (PDB code 1Z6T) were recovered to 100% confidence (10, 52). In agreement with earlier structural studies (12), high scoring matches (>99.4% confidence) were obtained with the Cdc6/Orc1 proteins of *Pyrobaculum aerophilum* (PDB 1FNN) and of *Aeropyrum pernix* in complex with DNA (PDB 2V1U). These proteins are members of a family of proteins involved in origin recognition and DNA replication in archaea and eukaryotes (45, 49, 53, 54). NB subdomain and tandem ARC domain residues (ARC1 and ARC2) of Rx1 are conserved between Cdc6/Orc1 of *A. pernix* and Rx1 (35.0% similarity and 12.7% identity between amino acids 134–479 of Rx1 and amino acids 13–382 of PDB entry 2V1U) (Fig. 1*A*).

Both the N-terminal NB and C-terminal ARC domain-like regions of Cdc6/Orc1 contact DNA, inducing deformation of the double helix (45, 49). The modeled tertiary structure of Rx1 (Fig. 1*B*, *left*) was related to Cdc6/Orc1 bound to DNA (PDB code 2V1U) (Fig. 1*B*, *center*) but differed from Cdc6/Orc1 in the DNA-unbound state (PDB code 1FNN) (Fig. 1*B*, *right*). An overlay demonstrated that the difference between the modeled tertiary structure of Rx1 and Cdc6/Orc1 in the DNA unbound state (PDB code 1FNN) was due to rotation of amino acids 279–388 of the C-terminal Cdc6/Orc1 ARC-like domain (Fig. 1*C*, *left* and *center*). Amino acids 279–388 of the Cdc6/Orc1 C-terminal ARC-like domain can be excised from PDB entry 1FNN and directly superimposed onto the Rx1 ARC2 domain to demonstrate how this rotation has occurred in the absence of any global structural change (Fig. 1*C*, *right*). Cdc6/Orc1 forms part of a larger family of structural homologues that includes the AAA+ ATPase SSO1545 from *Sulfolobus*, RuvB from *Thermus*, Orc2 from *Aeropyrum*, mammalian Apaf-1, CED-4 from *Caenorhabditis elegans*, and NLRC4 from mouse (9, 35, 55–61). These proteins all show a similar domain arrangement of an NB domain that is coupled via its neighboring ARC1 domain to a C-terminal ARC2 domain with varying orientations. For example, the individual domains of the closed form of mouse NLRC4 (PDB code 4KXF) can be extracted and superimposed onto Cdc6/Orc1 in the DNA-bound state (PDB 2V1U), although their actual orientation does not support a DNA binding activity. The modeled structural relationship with Cdc6/Orc1 suggests the intriguing possibility that Rx1 might also interact directly with DNA. We therefore investigated whether Rx1 is a DNA-binding protein.

##### Rx1 Binds Nucleic Acids in Vitro

**FIGURE 2.**
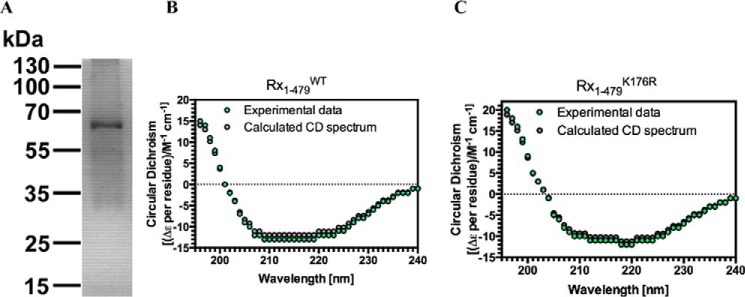
**Production and characterization of a recombinant Rx1 protein.** *A*, purified Rx1 protein (1.5 μg) was separated by 12.5% SDS-polyacrylamide gel electrophoresis and stained with Coomassie Blue. Molecular mass standards (in kDa) are indicated. Protein identity was confirmed by trypsin digest and MALDI-TOF analysis. Shown are a circular dichroism spectrum for Rx1(1–489)^WT^ (*B*) and Rx1(1–489)^K176R^ (*C*) depicting experimental data (*green dots*) and the spectrum calculated using CDSSTR (*gray dots*).

**FIGURE 3.**
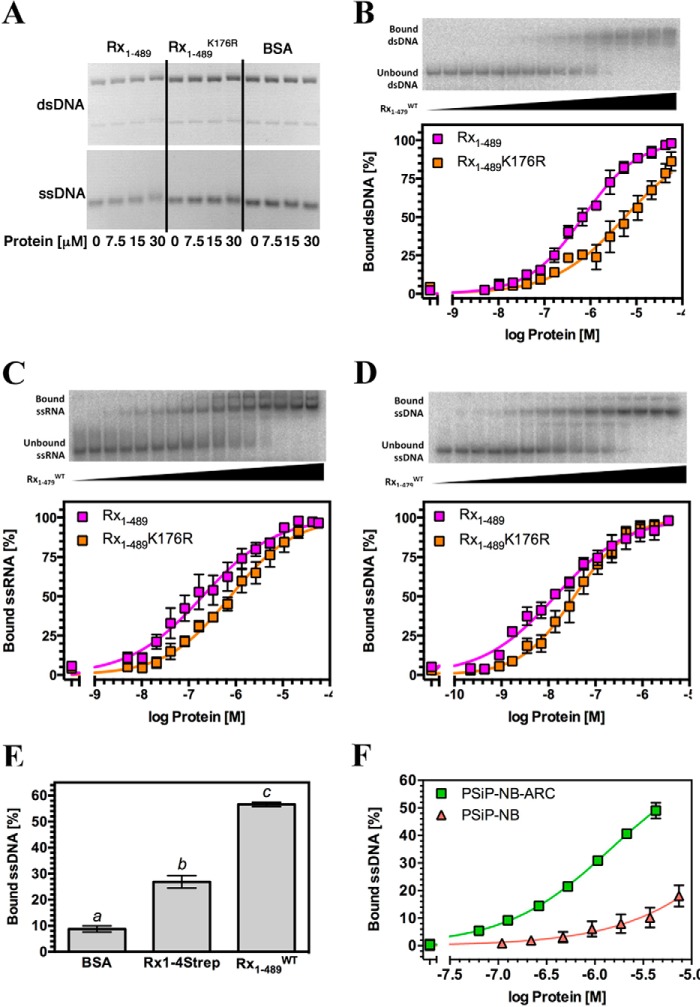
**The Rx1 CC-NBARC domains bind nucleic acids *in vitro*.** *A*, EMSA for Rx1(1–489)^WT^, Rx1(1–489)^K176R^, and BSA using 100 ng of φX174 virion DNA (ssDNA) or φX174 virion RF I DNA (dsDNA). For dsDNA, the *top band* represents relaxed circular DNA, whereas the *bottom band* represents supercoiled circular DNA. *B–D*, *top panels*, representative EMSA for Rx1(1–479)^WT^ showing raw data for binding to nucleic acids. *Bottom panels*, quantitative EMSA analysis giving apparent affinities of Rx1(1–489)^WT^ and Rx1(1–489)^K176R^ for dsDNA (*B*), ssRNA (*C*), and ssDNA (*D*) (means ± S.E. (*error bars*); *n* = 3–6). *E*, quantitative EMSA showing binding of 1 μm full-length plant-expressed Strep-tagged Rx1 (Rx1-4Strep), *E. coli* produced Rx1(1–489)^WT^, or BSA to ssDNA (means ± S.E.; *n* = 8; *bars* with *different letters* are significantly different (*p* < 0.05); one-way ANOVA with post hoc Tukey's multiple comparison). *F*, quantitative EMSA analysis giving comparative affinities of PSiP-NB-ARC and PSiP-NB for ssDNA (means ± S.E.; *n* = 3).

A possible direct Rx1-DNA interaction was investigated through *in vitro* experiments. EMSA using nucleic acid fragments of >5 kb derived from circular bacteriophage φX174 (62) represents a straightforward methodology to qualitatively assess interactions between a protein and either ssDNA or dsDNA with identical sequences. EMSAs were therefore performed using recombinant wild-type Rx1 protein (Rx1(1–489)^WT^), consisting of the CC-NB-ARC region but lacking the LRR domain (Fig. 2*A*). EMSA experiments performed with the Rx1(1–489)^WT^ protein showed an association with both ssDNA and dsDNA, producing a small upward shift in the migration of the nucleic acid that is fully consistent with similar EMSA experiments using unrelated DNA-binding proteins (Fig. 3*A*) (63). No mobility shift was observed with a control protein (BSA) that has a similar mass and isoelectric point as Rx1(1–489)^WT^.

The K176R mutation in the P-loop of Rx1 abolishes its ability to mount an immune response in the presence of the viral coat protein (23). The Rx1(1–489)^K176R^ loss-of-function mutant exhibited a barely detectable binding to DNA as compared with wild type Rx1 protein under these conditions (Fig. 3*A*). This difference is unlikely to be due to misfolding of the mutant because comparison of Rx1(1–489)^WT^ and Rx1(1–489)^K176R^ by circular dichroism (CD) reveals a generally similar secondary structure composition (Fig. 2, *B* and *C*). The CDSSTR method for secondary structure fraction prediction gave similar estimates for secondary structure content for both Rx1(1–489)^WT^ (61.6% helix, 14.8% sheet, 7.9% turn, 15.1% unresolved; normalized root mean square deviation = 0.066) and Rx1(1–489)^K176R^ (68.6% helix, 14.1% sheet, 7.9% turn, 8.7% unresolved; normalized root mean square deviation = 0.043) (64). Hence, subtle structural changes rather than an improperly folded protein probably explain differences in DNA binding between Rx1(1–489)^WT^ and Rx1(1–489)^K176R^.

The Rx1-DNA interaction was relatively stable because it could be visualized after gel electrophoresis (Fig. 3*A*). Nevertheless, although EMSA using circular bacteriophage φX174 DNA is a well established method to qualitatively assess protein-DNA interactions, it does not enable robust quantification of the affinity of a protein for nucleic acids. EMSA with small synthetic oligonucleotides is a standard method to quantify protein-nucleic acid interactions (65). Furthermore, the high molecular weight of φX174 DNA and consequent small band shifts were not suited to further analysis. We therefore quantified the affinity of Rx1(1–489) for various nucleic acids by EMSA using ^32^P-labeled synthetic oligonucleotides whose sequences were unrelated to that of bacteriophage φX174 DNA and which should provide more robust band shifts on EMSA due to their lower molecular weights (Fig. 3*A*). Rx1(1–489)^WT^ showed broadly similar apparent affinities (*K_d_*^app^) for dsDNA and ssRNA but exhibited a significantly higher apparent affinity for ssDNA (Fig. 3, *B–D*, and Table 1). The affinity of Rx1(1–489)^WT^ for dsDNA is within the submicromolar range and is of a similar magnitude as both eukaryotic and prokaryotic Cdc6/Orc1 proteins (66, 67). The apparent affinity of the Rx1(1–489)^K176R^ mutant for ssDNA, dsDNA, and ssRNA was lower than the apparent affinity of wild type Rx1 in each case, which corresponds to the observed lower affinity established using the φX174 DNA (Akaike information criterion, *p* > 0.99). To exclude the possibility that the observed nucleic acid binding was an artifact of the recombinant protein, we purified full-length Rx1 protein from plants. The protein was purified using a C-terminal 4-fold Strep-tag (Rx1-4Strep) from agroinfiltrated *N. benthamiana* leaves. The amount of purified Rx1 protein obtained was limited but sufficient to demonstrate that plant-derived Rx1-4Strep is also able to bind to ssDNA *in vitro* (Fig. 3*E*). Plant-derived Rx1-4Strep DNA binding was weaker than that of bacterially derived protein, which could be due to the fact that the majority of the full-length Rx1 is presumably in the autoinhibited off-state. Only a small fraction is proposed to be spontaneously active and thought to be responsible for the weak HR phenotype observed when Rx1 is overexpressed in the absence of the CP elicitor (68). In addition, the possibility cannot be excluded that the tag has impacted folding of a portion of the plant-expressed Rx1 protein.

**TABLE 1:** **Apparent dissociation constants for recombinant NLR domain interactions with nucleic acids** Values shown are the mean ± S.D. ND, not determined.

Protein	*K_d_*^app^ ssDNA	*K_d_*^app^ dsDNA	*K_d_*^app^ ssRNA
	μ*m*	μ*m*	μ*m*
Rx(1–489)^WT^	0.014 ± 0.002	0.70 ± 0.05	0.20 ± 0.03
Rx(1–489)^K176R^	0.036 ± 0.004	5.69 ± 0.85	0.77 ± 0.09
PSiP-NB	>50	ND	ND
PSiP-NB-ARC	4.08 ± 0.26	ND	ND

##### The NLR NB-ARC Domain Binds Nucleic Acids in Vitro

Despite the structural relationship between the Rx1 NB-ARC domains and Cdc6/Orc1 proteins, it is formally possible that the data of Fig. 3 can be explained by an interaction between nucleic acids and the N-terminal CC domain of Rx1(1–489) rather than its NB-ARC domain. We were unable to produce truncated Rx1 fragments encompassing solely the NB or NB-ARC domains. We therefore examined another plant NLR protein to assess whether the NB-ARC domain alone is able to bind nucleic acids and whether DNA binding is unique to Rx1 or represents a common property of at least a subset of plant NLRs. The NLR subdomains of the orphan NLR of the monocot *Zea mays* were chosen because both the NB and NB-ARC domains can be produced as soluble recombinant protein (14). We compared ssDNA binding of the NB subdomain of PSiP alone (PSiP-NB) with that of the complete NB-ARC domain of PSiP (PSiP-NB-ARC) (Fig. 3*F*). Although both fragments bound, the PSiP-NB-ARC domains bound ssDNA with a considerably higher affinity than the PSiP-NB domain alone (Table 1). Together, these data demonstrate that the NB-ARC domain is sufficient for nucleic acid binding in Rx1 and PSiP, that DNA binding is a property of at least a subset of plant NLR proteins, and that both the NB and the ARC subdomains contribute to the DNA interaction.

##### Rx1 Deforms DNA

**FIGURE 4.**
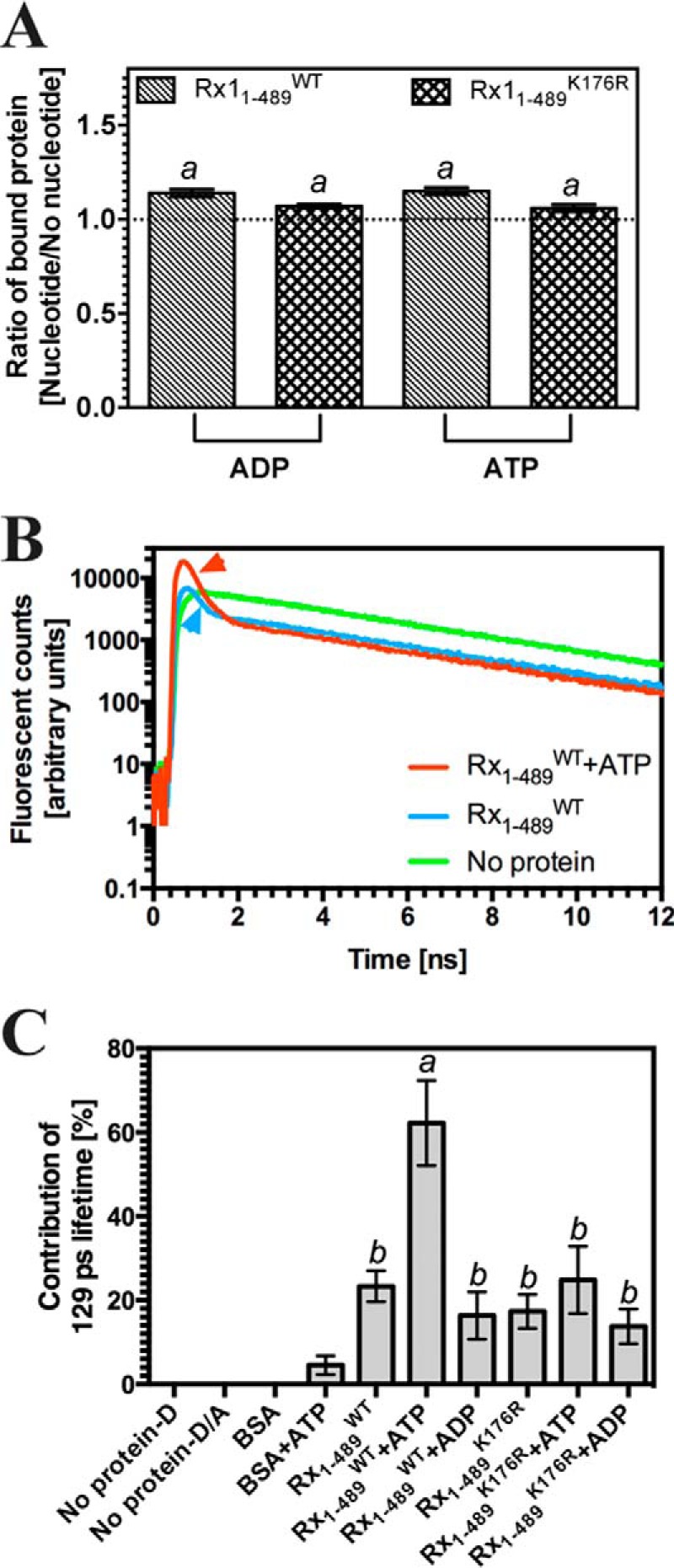
**Rx1(1–489) bends dsDNA.** *A*, double-stranded DNA-binding by Rx1(1–489)^WT^ and Rx1(1–489)^K176R^ assessed by EMSA plotted as a ratio of binding in the presence of 1 μm nucleotide compared with no nucleotide (means ± S.E. (*error bars*); *n* = 3; *a*, *p* > 0.05). The DNA used is identical to that used for Fig. 3*B. B*, sample time-resolved data for a control (*No protein*) and Rx(1–489)^WT^ with and without ATP. The data show fluorescent counts from the fluorescent donor plotted against time. The appropriately *colored arrowhead* indicates the 129-ps lifetime associated with energy transfer. *C*, the percentage contribution of the 129-ps lifetime for fluorescent donor in the presence of BSA or Rx1(1–489) and nucleotides (*D*, donor-labeled oligonucleotide only, no protein; *D/A*, donor- and acceptor-labeled oligonucleotide, no protein) (means ± S.E.; *n* = 3–11; *bars* with *different letters* are significantly different (*p* < 0.05); one-way ANOVA with post hoc Tukey's multiple comparison).

In the “switch” model for plant NLR activation, binding of ATP to the NB-ARC domain establishes the “on” state, whereas hydrolysis of ATP to ADP restores the “off” state (9). An intact P-loop is essential for nucleotide binding, and mutations in this motif typically result in loss-of-function alleles (9). We therefore investigated the relationship between P-loop-dependent ATPase activity and DNA binding. We detected no ATPase activity in Rx1(1–489)^WT^, possibly indicating the absence of a catalytic water molecule, as observed previously for the STAND ATPase Ced-4 (69). Neither ATP nor ADP had any discriminatory influence on Rx1(1–489) binding to dsDNA (Fig. 4*A*). We therefore investigated whether Rx1 has activities at DNA other than binding that are affected by the type of nucleotide (ATP/ADP) bound. The Cdc6/Orc1 family proteins ORC1 of *A. pernix* and the Orc1-1/Orc1-3 heterodimer of *S. solfataricus* substantially deform origin DNA by bending it with angles of 35 and 20°, respectively, thereby inducing localized melting of the double helix (45, 49, 70). We therefore examined whether Rx1(1–489) can deform DNA in a similar fashion and whether this process is nucleotide type-dependent. To measure DNA bending, time-resolved FRET was used because it allows measurements of distances between fluorophores. This method offers considerable advantages over steady-state FRET because the fluorescence lifetime represents an intrinsic property of the fluorophore and is independent of concentration, photobleaching, or light scattering (71). We monitored DNA deformation using time-resolved FRET with dual end-labeled dsDNA (72). Upon FRET, the fluorescence lifetime of the donor fluorophore decreases; therefore, we deconvoluted the fluorescence donor emission for its constituent lifetime components. We hypothesized that following DNA bending, we would observe a shortened donor lifetime due to energy transfer to the acceptor. DNA bending was assessed under conditions to saturate binding of Rx1(1–489)^WT^ or Rx1(1–489)^K176R^ to dsDNA. Bending was evident as a decrease in the contribution of a 4.1-ns component (indicative of unperturbed donor emission; fluorescein fluorescence) to and the appearance of a 129-ps component during the total fluorescence decay of FRET donor emission (Fig. 4*B*, the 129-ps component is marked with an *arrow*). The 129-ps lifetime component, attributed to energy transfer, was only observed for the Rx1(1–489)^WT^ protein and Rx1(1–489)^K176R^ protein and not in controls without protein or with BSA except for a minor contribution with the latter in the presence of ATP (Fig. 4*C*). The 129-ps lifetime corresponds to a calculated donor-acceptor distance of 29 Å (assuming isotropic orientations) and therefore an overall bend angle of 42° around a presumed oligonucleotide midpoint.

Next, it was investigated whether nucleotides had an influence on the observed DNA bending. Notably ATP, but not ADP, strongly increased the contribution of the 129-ps lifetime to the overall time-resolved data. This increase was only observed for the Rx1(1–489)^WT^ protein and not for the Rx1(1–489)^K176R^ mutant, indicating that DNA bending requires an intact P-loop capable of binding nucleotides. The distinct response of the Rx1(1–489)^WT^ protein following incubation with either ADP or ATP provides additional support for a correct native fold of the nucleotide-binding pocket in the recombinant protein. The absence of any change in the value of the shortened lifetime (129 ps) shows that the calculated donor-acceptor distance is constant. Because the relative proportion of the 129 ps lifetime to the total fluorescence signal increases in the presence of ATP and Rx1(1–489)^WT^, we can conclude that ATP binding enhances the pool of protein-DNA complexes in the bent state but not the bending angle.

**FIGURE 5.**
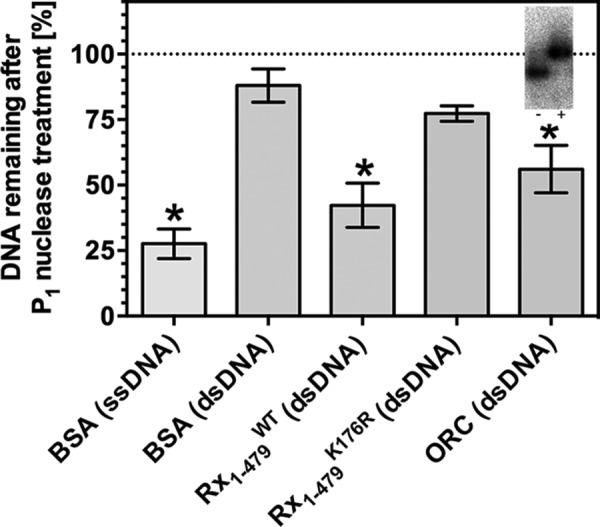
**Rx1(1–489) induces localized DNA melting.** DNA remaining undigested after treatment with P_1_ nuclease in the presence of BSA, Rx1(1–489)^WT^, Rx1(1–489)^K176R^, or ORC as a percentage of total DNA (means ± S.E. (*error bars*); *n* = 6–19; *, *p* < 0.05 compared with dsDNA in the presence of BSA by one-way ANOVA with post hoc Dunnett test). The *inset* shows a control EMSA using the C3/mORB dual site DNA sequence at *oriC2* of *S. solfataricus* in the presence or absence of 1.5 μm ORC.

Time-resolved FRET is a well validated method to examine intramolecular distances and therefore DNA topology, but it does not provide further information on other DNA distortions associated with changes in topology. To examine whether Rx1(1–489) can induce local DNA melting, as has been observed for Orc1, we explored a non-fluorescence-based methodology. P_1_ nuclease has been used previously as a tool to examine local DNA distortion using the Orc1 protein of *A. pernix* (70). We therefore examined the sensitivity of dsDNA oligonucleotides to the ssDNA-specific P_1_ nuclease in the presence of Rx1(1–489) (73). As expected, ssDNA was significantly degraded by P_1_ nuclease (positive control), whereas dsDNA, in the presence of BSA (negative control), was largely resistant to P_1_ nuclease activity (Fig. 5). Although dsDNA was more sensitive to P_1_ nuclease in the presence of Rx1(1–489), the mutant Rx1(1–489)^K176R^ did not induce local DNA melting because no increased DNA degradation was observed. Thus, although in the *absence* of nucleotides, Rx1(1–489)^K176R^ bends dsDNA to a similar magnitude as Rx1(1–489)^WT^, its failure to melt DNA might be a manifestation of subtle changes to DNA binding evidenced through the decrease in binding affinity (Fig. 3, *B–D*). These experiments were insufficiently sensitive to examine the further influence of nucleotides on NLR-mediated DNA melting. The P_1_ sensitivity of dsDNA in the presence of Orc1-1/Orc1-3 was indistinguishable from that of dsDNA in the presence of Rx1(1–489)^WT^, supporting the interpretation that plant NLRs can cause local dsDNA melting. In conclusion, Rx1 is able to both bend DNA and provoke local DNA melting, and this bending activity requires an intact P-loop and is stimulated by the presence of ATP.

##### Rx1 Preferentially Binds Specific DNA Topologies in Vitro

**FIGURE 6.**
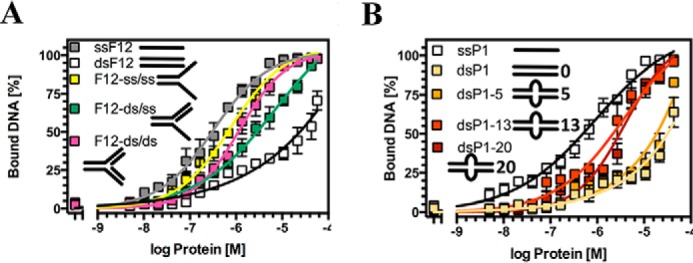
**Rx1 preferentially binds distorted DNA topologies.** *A*, quantitative EMSA analysis giving comparative affinities of Rx1(1–489)^WT^ for ssDNA (*ssF12*), dsDNA (*dsF12*), branched dsDNA with two dsDNA arms (*F12-ds/ds*), branched dsDNA with two ssDNA arms (*F12-ss/ss*), and branched dsDNA with one ssDNA and one dsDNA arm (*F12-ds/ss*) (means ± S.E. (*error bars*); *n* = 3–4). *B*, quantitative EMSA analysis giving comparative affinities of Rx1(1–489)^WT^ for ssDNA (*ssP1*), dsDNA (*dsP1*), and dsDNA with bubbles of varying sizes (means ± S.E.; *n* = 3–4).

We sought independent experimental support for Rx1-mediated distortion of DNA. We hypothesized that if Rx1 distorts linear DNA, then the free energy of Rx1 binding to DNA structures that resemble the distorted state would be favored (with a corresponding increase in affinity). Indeed, Rx1 bound branched double-stranded DNA with a significantly higher affinity than control linear double-stranded DNA of similar sequence (Fig. 6*A*; compare *dsF12* with *F12-ds/ds*). The branched double-stranded DNA represents a non-natural DNA and is a control to demonstrate a preference for Rx1(1–489) binding to a branched topology. When comparing binding affinities for naturally occurring branched topologies, we noted a higher affinity for branched structures with one dsDNA and two ssDNA arms (*e.g.* similar to a transcription bubble) compared with structures with one or two duplex arms (*e.g.* resembling a DNA replication fork) (Fig. 6*A*, compare *F12-ds/ss* with *F12-ss/ss*). Consistent with our model of local DNA melting, Rx1(1–489) showed a higher affinity for small DNA bubbles compared with linear dsDNA (Fig. 6*B*). This increased affinity was not due to the increased affinity for ssDNA because affinity did not correlate with increasing DNA bubble size. Although these data cannot reveal the exact nature of the distorted DNA state on DNA bending (Fig. 4) and melting (Fig. 5), analysis of the relative affinities does demonstrate that Rx1 shows an increased affinity for specific DNA structures, and the DNA distortion we observed in the presence of Rx1 is probably a genuine response following its activation.

##### Rx1 DNA Binding Is Specifically Activated by Its Cognate Elicitor in Vivo

DNA binding, bending, and melting is a new aspect of plant NLR biochemistry. To validate DNA as a downstream target for NLR signaling and link this biochemical activity to its function in plant cells, we tested whether Rx1 is able to interact with DNA *in vivo*. To investigate the possibility of a direct interaction with genomic DNA inside the cell, we studied Rx1-DNA interactions in the nucleus using Forster resonance energy transfer-fluorescence lifetime imaging microscopy (FRET-FLIM). FRET-FLIM has been used previously to demonstrate transcription factor binding to DNA in response to environmental signals (74).

**FIGURE 7.**
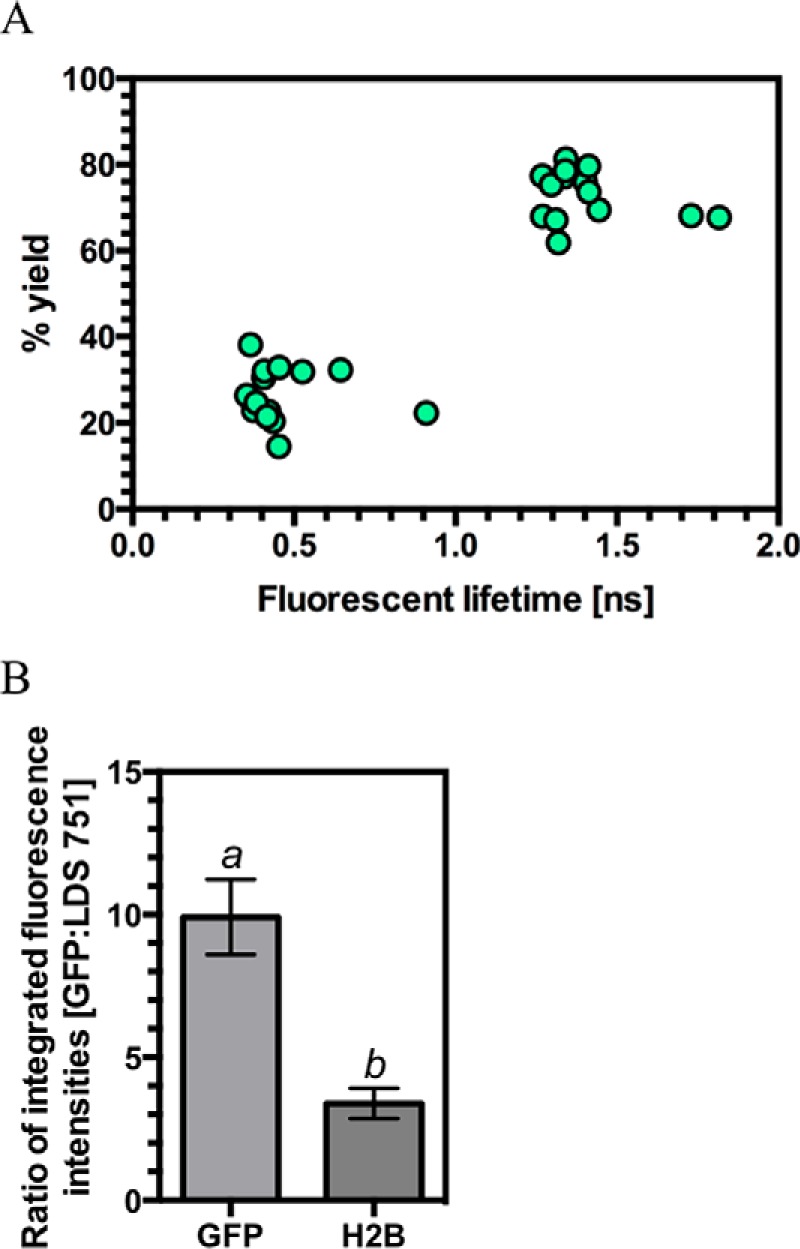
**Individual fluorescent lifetime signals for GFP can be resolved from agroinfiltrated plants.** *A*, plot of the identified fluorescent lifetimes for GFP from agroinfiltrated *N. benthamiana* epithelial cell nuclei against the percentage yield for that lifetime. The graph represents 14 measurements from seven independently infiltrated leaves with each measurement providing two fluorescent lifetime values. *B*, ratios for the integrated emission intensities for GFP and LDS 751 in GFP (negative control) and H2B-GFP (positive control) agroinfiltrated *N. benthamiana* (*n* = 6–7; *bars* with *different letters* are significantly different (*p* < 0.05); Student's *t* test). *Error bars*, S.E.

**FIGURE 8.**
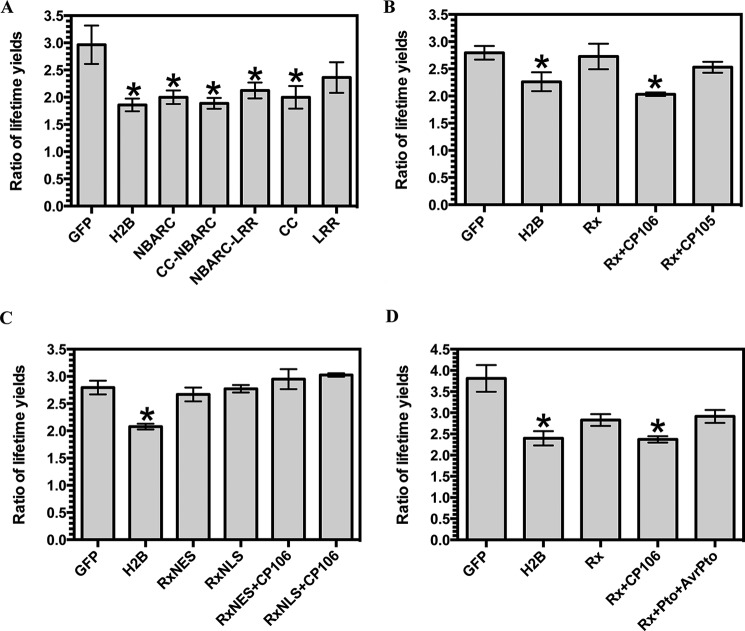
**Binding of Rx1 protein domains to DNA *in situ*.** *A*, the ratio of the long (>1.0 ns) to short (<0.5 ns) GFP lifetimes for GFP fusion constructs representing varying Rx1 subdomains produced in *N. benthamiana* leaves using agroinfiltration (*n* > 6; *, *p* < 0.05 compared with GFP by one-way ANOVA with post hoc Dunnett test). *B*, ratio of the long (>1.0 ns) to short (<0.5 ns) GFP lifetimes for Rx1-GFP full-length constructs alone and upon co-expression with virulent (CP105) and avirulent alleles (CP106) of the PVX CP (*n* > 6; *, *p* < 0.05 compared with GFP by one-way ANOVA with post hoc Dunnett test). *C*, ratio of the long (>1.0 ns) to short (<0.5 ns) GFP lifetimes for GFP-NLS-Rx1 and GFP-NES-Rx1 full-length constructs alone and upon co-expression with the avirulent allele of PVX, CP106 (*n* = 4; *, *p* < 0.05 compared with GFP by one-way ANOVA with post hoc Dunnett test). *D*, ratio of the long (>1.0 ns) to short (<0.5 ns) GFP lifetimes for Rx1-GFP full-length construct alone and upon co-expression with either the avirulent allele of PVX, CP106, or the Pto kinase and AvrPto (*n* = 12–20; *, *p* < 0.05 compared with GFP by one-way ANOVA with post hoc Dunnett test). *Error bars*, S.E.

*N. benthamiana* was infiltrated with *A. tumefaciens* carrying constructs encoding either GFP (negative control), a protein consisting of *A. thaliana* histone H2B fused to GFP (GFP-H2B; positive control), or discrete domains of Rx1 fused to GFP. Previous work has similarly utilized H2B-GFP and naked GFP as controls for DNA binding in paraformaldehyde-fixed preparations (74). The constituent fluorescence lifetimes for the GFP tag were examined in leaves counterstained with LDS 751. LDS 751 is a cell-permeable nucleic acid stain with an excitation maximum, when bound to DNA, that overlaps with the GFP emission spectrum. GFP showed two distinct lifetimes at ∼0.5 and 1.5 ns (Fig. 7*A*). Because energy transfer from donor (GFP) to acceptor (LDS 751) decreases the fluorescent lifetime, we hypothesized that the shorter lifetime for GFP is representative of energy transfer consistent with an interaction between the fluorophores. Notably, such a decrease in the GFP fluorescence lifetime by time-correlated single-photon counting is independent of protein expression levels, quenching, photobleaching, or fluctuations in the excitation source. A decrease inlifetime can therefore specifically be attributed to quenching of the excited state of the GFP and represents strong evidence for energy transfer from GFP to LDS 751 and thus a direct protein-DNA interaction. Consistent with this interpretation, a significant decrease in the ratio of the yields of the GFP fluorescence lifetimes was observed for the DNA-binding protein GFP-H2B (Fig. 8*A*). To demonstrate that energy transfer to LDS 751, and not to surrounding proteins, explains the data, we confirmed that the decrease in the fluorescence GFP lifetime ratio indicative of DNA binding was correlated with an increase in LDS 751 emission that does not arise from the excitation source (Fig. 7*B*). Although the exact stoichiometry of GFP and LDS 751 levels is not known in each experiment, the finding that the ratio of fluorescence emission for GFP/LDS 751 is significantly decreased for H2B compared with the negative control is strong evidence that the reduction in GFP lifetimes is due to energy transfer to LDS 751 and not to an alternative molecule. As predicted, Rx1-GFP fusions containing the NB-ARC domain (NB-ARC-GFP, CC-NB-ARC-GFP, and NB-ARC-LRR-GFP) showed a significant decrease in the ratio of GFP lifetime yields, consistent with its observed DNA binding activity *in vitro*, whereas the LRR (GFP-LRR) domain did not. Surprisingly, the CC domain alone (CC-GFP) also showed a decrease in the ratio of GFP lifetime yields. The Rx1 CC domain has been shown previously to associate with a high molecular weight complex in the nucleus (23), and our findings indicate that this complex probably contains genomic DNA. Taken together, these data demonstrate that the CC and CC-NB-ARC Rx1 domains can form a stable interaction with DNA *in situ*. The FRET-FLIM methodology used is independent of expression levels of the various constructs. However, the methodology can be sensitive to cleavage of the GFP tag of even a small percentage of expressed protein. Fortunately, cleavage of the GFP tag can be resolved because it yields a *high* rather than the observed *low* ratio of fluorescence lifetimes, allowing us to conclude that the positive results for DNA binding *in situ* are not attributable to tag cleavage.

Next, we investigated whether the full-length Rx1 molecule (Rx1-GFP) also binds to DNA in the plant cell. Notably, a full-length Rx1-GFP fusion showed no binding to DNA as compared with the negative control (Fig. 8*B*). This implies that the inactive full-length Rx1 protein adopts a structure refractory to interacting with DNA. To test whether there is a relationship between DNA-binding and Rx1 activation and subsequent immune signaling, we next co-expressed Rx1 with the PVX coat protein elicitor, which is known to trigger immunity (23). Full-length Rx1-GFP was found to bind to DNA only in the presence of the wild type (avirulent) coat protein (CP106) and not in the presence of a mutant (virulent) elicitor (CP105) that is unable to activate Rx1 (Fig. 8*B*). These data show that DNA binding *in vivo* by Rx1 only occurs upon perception of its cognate elicitor.

To test whether DNA binding *in situ* requires elicitor recognition in the cytoplasm, we investigated DNA binding of Rx1-GFP fused to either an NLS or an NES. These chimeric tags have been demonstrated previously to constrain Rx1 to the nucleus or cytoplasm, respectively (23). This experiment addresses two questions. 1) Is enforced nuclear accumulation of a GFP fusion protein sufficient to confer DNA binding? 2) At what subcellular localization can Rx1 be activated by the coat protein to permit DNA binding? GFP-NLS-Rx1 did not bind DNA in the presence or absence of CP106, demonstrating that enforced Rx1 accumulation in the nucleus is insufficient to drive DNA binding *and* that DNA binding requires CP106 recognition in the cytoplasm, consistent with previous findings (23) (Fig. 8*C*). GFP-NES-Rx1 also did not bind DNA in the presence or absence of CP106, demonstrating that the DNA binding signal is dependent on the ability of the cytosolic Rx1 protein to gain access to genomic DNA regardless of exposure to CP106. GFP-NES-Rx1 and GFP-NLS-Rx1 are not sensitive to cleavage of GFP, thus excluding the possibility that the observed absence of DNA binding is due to sensitivity to proteolysis (23). Taken together, the data support a model where Rx1 binding to DNA is a specific nuclear event in immune activation subsequent to coat protein detection in the cytosol.

We further investigated binding of Rx1-GFP to DNA upon activation of another immune receptor to exclude the possibility that Rx1 binds DNA as a nonspecific consequence of defense activation. We co-infiltrated *N. benthamiana* with *A. tumefaciens* carrying constructs encoding Rx1-GFP, the Pto kinase of tomato, and the AvrPto effector. The Pto kinase activates an immune response in *N. benthamiana* upon binding the AvrPto effector of *Pseudomonas syringae* pv. tomato (75–77). Rx1-GFP did not bind DNA when Pto was activated by AvrPto, indicating that Rx1-GFP DNA binding is not a generic response following defense activation (Fig. 8*D*). Because the role of Rx1 in immunity is dependent upon both its activation by the viral coat protein in the cytoplasm and its DNA binding activity in the nucleus, our findings therefore provide the first evidence for a direct molecular target between activation of a plant NLR and subsequent cellular immune responses.

## Discussion

The molecular mechanism underlying the function of activated NLR proteins in plant immunity is a crucial, but still unanswered, question. Existing *in vitro*, *in vivo*, and bioinformatics data pinpoint the NB-ARC domain as a central switch in regulating NLR activity. We here propose that the NB-ARC domain also possesses an intrinsic DNA binding activity, and we demonstrate that its DNA binding activity is associated with the cellular immune response. The Rx1 protein is observed to bind and deform dsDNA *in vitro* and to bind cellular DNA in response to activation following elicitor perception. Importantly, although the described biochemistry for Rx1 is novel for a NLR protein, DNA distortion is a well characterized feature of other proteins that interact with DNA through non-sequence-specific interactions, including TATA box-binding protein (78, 79), integration host factor (80), and the HMG box (81). Rx1 biochemistry is therefore consistent with the activity of known DNA-binding proteins.

Our observation that Rx1 can interact with DNA in response to immune activation might provide a rationale for its nuclear localization. For example, a P-loop mutant in Rx1 can potentially establish a correlation between DNA binding and immunity. The K176R P-loop mutant of Rx1 is defective in triggering immunity (23) to PVX. We show that this mutant is also defective in nucleotide-dependent DNA bending and DNA melting *in vitro*. This finding represents a potential link between the ability of Rx1 to distort DNA *in vitro* and the ability to trigger immunity *in planta*. Equivalent mutations in the NB domain of Cdc6 have been used to investigate the activity of Cdc6 at dsDNA (82, 83).

*In vivo* activation of Rx1 by the PVX coat protein induces the plant immune response (84) (Fig. 8*B*). We found that Rx1 only bound nuclear DNA following recognition of the CP106 coat protein and not the CP105 variant, which is unable to trigger Rx1 signaling. These data show that only properly activated Rx1 has the ability to interact with DNA *in situ*. In addition, only cytosolic recognition of CP106, followed by translocation of activated Rx1 to the nucleus, results in full activation of immunity (23). We demonstrate that, even in the presence of the CP106 coat protein, no DNA binding occurs when Rx1 is artificially retained in either cytosol or nucleus (Fig. 8*C*). This finding presents a potential link between the known spatial requirements for Rx1-mediated immune activation and the DNA binding observed *in situ*. Such a translocation mechanism might be analogous to that of WHIRLY1, an immune activated transcriptional regulator that translocates to the nucleus and is involved in defense gene expression (85). *In vitro*, full-length (hence mostly inactive) Rx1 purified from *N. benthamiana* did interact with DNA, albeit less strongly than the CC-NBARC form produced in *E. coli* (Rx1(1–489)^WT^) (Fig. 3*E*), which is free of the autoinhibitory constraint posed by the LRR domain (86). DNA binding *in vitro* with full-length Rx1 occurred under conditions where relatively high protein concentrations can be assayed. Presumably, Rx1 levels *in vivo* are too low to observe DNA binding in its non-activated state (Fig. 8*B*). The observed DNA binding by full-length Rx1 *in situ* is not a generic consequence of plant immunity because activation of immunity through another immune receptor (Pto/AvrPto) did not induce Rx1 DNA binding. We therefore propose that DNA binding by Rx1 upon PVX coat protein perception is an essential, specific, and early step in the cellular immune response.

The Rx1 NBARC domains share remarkable biochemical properties with the Cdc6/Orc1 family DNA-binding proteins. Rx1 was observed to bind both ssDNA and dsDNA similar to ORC of *Saccharomyces cerevisiae* (87). The Cdc6/Orc1 homology with NLR proteins and the DNA binding characteristics of the separate PSiP NB and NB-ARC domains accord with multiple contacts with DNA across both NB and ARC domains. Hence, single point mutations are unlikely to abolish DNA binding, and, consistent with previous observations (67), we have not identified point mutations that ablate DNA binding. Eukaryotic ORCs lack DNA sequence specificity *in vitro* but show higher affinity for specific DNA topologies (88, 89). Consistent with this, Rx1 shows higher affinity for branched and melted DNA topologies than for dsDNA. The bend angle introduced into DNA by Rx1(1–489) is also of a magnitude similar to that observed in crystal structures of ORC1 from *A. pernix* (45). Analysis of *A. pernix* ORC2 revealed a considerable conformational flexibility stabilized by ATP (54). In this context, it is interesting to note that although the bend angle is identical for both wild type and mutant Rx1 proteins in the absence of nucleotide or presence of ADP, the *population* of DNA in the bent state was more prevalent in Rx1(1–489)^WT^ supplemented with ATP. The Rx1 activated state is therefore specifically linked to DNA distortion.

The activity of Rx1 on DNA provides biochemical evidence that Rx1 might act as a transcriptional regulator through its NB-ARC domain. DNA binding by a NLR is a signaling event because the NB-ARC domain is not involved in recognition specificity. Pathogen recognition by NLRs is typically determined by the LRR, often in conjunction with integrated effector targets (90, 91). A key process in transcriptional activation is the distortion of DNA to enable the formation of the transcription preinitiation complex (92–95). In the cell, Rx1 protein might have sequence-specific DNA binding conferred by interacting protein partners, whereas the NB-ARC domain distorts DNA to a state that activates or represses transcription, depending upon the locus (96). The region encompassing the CC domain, which might interact with DNA via an accessory protein (*e.g.* a transcription factor), could confer this sequence specificity. The identification of such a binding partner that can confer sequence specificity to the Rx1-DNA interaction represents a significant challenge for the future.

In summary, we have identified a conserved DNA binding and distorting activity in the NB-ARC domain of the Rx1 protein *in vitro* and link Rx1 activation following elicitor recognition to nuclear DNA binding *in situ*. Rx1 induces cellular immune responses after viral coat protein recognition. We hypothesize that a function for Rx1 is to manipulate DNA into an “immune competent” state. The precise nature and role of this Rx1 protein-DNA immune competent state can now be addressed in future studies.

## Author Contributions

S. F., P. D. T., C. H. D., G. B. S., A. S. E. C., E. J. S., L. B. W., and F. K. K. G. performed the experiments. S. F., P. D. T., C. H. D., G. B. S., E. J. S., G. J. S., A. G., L.-O. P., F. L. W. T., and M. J. C. analyzed the data. M. R. K., G. J. S., A. G., L.-O. P., F. L. W. T., and M. J. C. conceived the experiments. M. J. C. and F. L. W. T. conceived the overall project and wrote the manuscript. All authors reviewed the results and approved the final version of the manuscript.

## Footnotes

3 The abbreviations used are:

NLR: nucleotide-binding leucine-rich repeat
AAA+: ATPases associated with diverse cellular activities
CC: coiled-coil
FLIM: fluorescence lifetime imaging microscopy
NB: nucleotide-binding
NB-ARC: nucleotide-binding, Apaf-1, R-proteins, and CED-4
LRR: leucine-rich repeat
NES: nuclear export sequence
NLS: nuclear localization sequence
PSiP: pollen-signaling protein
PVX: protein virus X
STAND: signal transduction ATPases with numerous domains
TIR: Toll-interleukin 1 receptor
Bistris propane: 1,3-bis[tris(hydroxymethyl)methylamino]propane
ANOVA: analysis of variance
PDB: Protein Data Bank.
